# Ants determine their next move at rest: motor planning and causality in complex systems

**DOI:** 10.1098/rsos.150534

**Published:** 2016-01-13

**Authors:** Edmund R. Hunt, Roland J. Baddeley, Alan Worley, Ana B. Sendova-Franks, Nigel R. Franks

**Affiliations:** 1 School of Biological Sciences, University of Bristol, Life Sciences Building, 24 Tyndall Avenue, Bristol BS8 1TQ, UK; 2 School of Experimental Psychology, University of Bristol, 12a Priory Road, Bristol BS8 1TU, UK; 3 Department of Engineering Design and Mathematics, University of the West of England, Frenchay Campus, Coldharbour Lane, Bristol BS16 1QY, UK

**Keywords:** movement, motor planning, self-similarity, division of labour, intermittent top-down causality, complex social systems

## Abstract

To find useful work to do for their colony, individual eusocial animals have to move, somehow staying attentive to relevant social information. Recent research on individual *Temnothorax albipennis* ants moving inside their colony’s nest found a power-law relationship between a movement’s duration and its average speed; and a universal speed profile for movements showing that they mostly fluctuate around a constant average speed. From this predictability it was inferred that movement durations are somehow determined before the movement itself. Here, we find similar results in lone *T. albipennis* ants exploring a large arena outside the nest, both when the arena is clean and when it contains chemical information left by previous nest-mates. This implies that these movement characteristics originate from the same individual neural and/or physiological mechanism(s), operating without immediate regard to social influences. However, the presence of pheromones and/or other cues was found to affect the inter-event speed correlations. Hence we suggest that ants’ motor planning results in intermittent response to the social environment: movement duration is adjusted in response to social information only between movements, not during them. This environmentally flexible, intermittently responsive movement behaviour points towards a spatially allocated division of labour in this species. It also prompts more general questions on collective animal movement and the role of intermittent causation from higher to lower organizational levels in the stability of complex systems.

## 1. Introduction

### 1.1 Complex systems and the movement of their parts

One of the typical characteristics of complex systems (especially in biology) is that they have several levels of organization, forming a hierarchy of systems and sub-systems [1]. An ant colony is a paradigmatic example. The highest level of organization is the colony, composed of hundreds or thousands of workers (up to 200–400 in the case of our study species *Temnothorax albipennis* [2]). In turn each individual ant is composed of many cells—with, for example, the ant brain being composed of 10^5^–10^6^ neurons (the honeybee *Apis mellifera* suggests an upper limit of 10^6^ [3]). Emergence of cognition at the level of the individual and at the colony (‘collective cognition’, for instance in decision-making [4]) depends on robustness in the order that arises from interactions among parts at a lower level [1]. In the case of ants, the interactions between the parts of the colony are often indirect, relying on modification of the local environment, for instance through pheromones, a coordination mechanism referred to as stigmergy [5].

A concept related to emergent hierarchies of organization is that of top-down causation. A reductionist view of biology would see ant colony decisions as derivable from individual choices, which are then in turn derivable from processes at smaller scales down to the genes (bottom-up causation). In reality, there is no privileged level of causation [6], with the dynamics of colony-level behaviours exercising a downward influence on the behaviour of individuals, for instance, so that feedback between different organizational levels takes place, and on many different time and length scales. This often relies on the exchange of information between the system and its environment, as in the case of stigmergic communication.

Fundamentally, the adaptability and persistence of complex social systems like ant colonies relies on the parts at an intermediate level of organization—the workers, at the level of the individual—being able to *move* from one place to another. This is so they can respond and adapt to a changing physical and social environment. Therefore, understanding how movements of different speeds and durations are planned and executed by organisms in different physical and social environments is of critical importance to understanding the functioning of complex social systems more generally. This includes the identification of the most important causal links between organizational levels and their mode of operation.

Research that tracked individual ants moving inside their colony’s nest [7] found a power-law relationship between a movement’s duration and its average speed; and a universal speed profile for movements short and long showing that they mostly fluctuate around a constant average speed. From this predictability it was inferred that movement durations are somehow determined before the movement itself. This finding is not confined to insects: recent research on human motor activity, which had participants wear a wristwatch-sized accelerometer for around a month, also found universality in motor activity fluctuations, again implying some degree of predetermination to a motion’s duration in a social context [8]. Past functional magnetic resonance imaging research on humans working alone in a binary choice experiment also found predictability from brain activity patterns up to 10 s before the decision entered their consciousness [9]. To determine the role of social interaction in generating self-similar, predictable movement behaviour in a complex system we track and analyse the movement patterns of 33 individual ants exploring outside the nest for a cumulative time of over 24 h. Our findings suggest that top-down causation from the colony-level of organization does not act continuously on individuals.

### 1.2 Movement in ant colonies

Ant societies are remarkably successful, accounting for 15–20% of the terrestrial animal biomass on average, and up to 25% in tropical regions [10]. An important factor behind their flexibility and resilience may be the evolution of robust, scalable movement algorithms that allow worker ants to regulate suitably their activity as they serve their colony, undertaking work and searching for new tasks in a variety of different physical and social environments. For instance, ants generally begin life with inside the nest work such as brood care before shifting to outside the nest work such as foraging; the European wood ant (*Formica polyctena*) offers a typical example [11]. About half of the worker’s time is spent at rest while in the other half it is engaged in some social activity or foraging [12]. Inter-individual variation in periods of activity in worker ants is typical, with in some cases a subset of ants doing consistently less work than their nest-mates, perhaps functioning as reserve workers ready to tackle large tasks [13]. Necessity would also seem to dictate that individual ants have an inherent flexibility to seek out and perform different tasks in the upkeep of their colony, owing to hazards such as predation, to make it robust to the loss of sections of workers; in their self-organized society there is no central authority to redirect workers to new tasks [14]. This task flexibility also demands that a range of associated movements (short and long, fast and slow) can be efficiently planned and executed, while also achieving an effective colony-level division of labour. Understanding how the movement patterns of ants are similar or different when they are outside their nest in the wider world, compared with how they behave inside the nest, should provide new insight into how work in their complex social system is organized, and shed light on emergent patterns of colony-level activity [15,16].

The activity of individual ants is sporadic, with periods of movement punctuated by periods of rest. Attempting to make generalizations about these periods of activity is difficult, not least because eusocial insects like ants live in the presence of what may be many hundreds of fellow colony members. The challenge is made still greater by inter-individual variation associated with differences in maturity or experience [17], and the presence of scale invariant spontaneous activity [18–22]. Nevertheless, by employing techniques more commonly used in statistical physics, recent research on the activity of ants inside their colony’s nest uncovered the existence of a universal relationship between the duration of an activity event (defined as a sequence of non-zero speeds bounded by zero speeds) and its average speed [7]. First, it was discovered that the average speed of an ant’s activity event is higher when event duration is longer, and that statistically this follows a power-law relationship of the form 〈*v*(*T*)〉_*t*_=*aT*^*β*^, where 0<*β*<1 (average speed increases sub-linearly with event duration). Second, the average speed profiles for different event durations can be rescaled to fit onto the same universal scaling function. This showed an underlying similarity to movement events inside the nest, with the average speed associated with a given event duration being maintained for most of the movement time, apart from short periods of acceleration and deceleration at the beginning and the end. Since the beginning of the movement thus contains information about the event’s eventual duration (via the quickly attained average speed and the power-law relation), it was concluded that activity event durations must be determined before commencement to some degree. Experimental treatments varying the physical size of the nest containing the ant colony showed that the shape of the scaling function was unchanged. However, some degree of behavioural change in response to altered physical space was found because the *β* exponent was larger in the bigger nest size. This may also be adduced as further evidence for event duration predetermination, because for a given speed, durations are longer in smaller nests that have more densely spaced colony members and brood items, which could interrupt an ant’s movement. This seems to rule out causality in the opposite direction (from higher speeds to longer durations).

The movement of ants within their colony’s nest were thus shown to be flexible in adjusting to the available physical space, and also predictable in terms of the average speed profile over the course of the event, and in the event’s consequent duration. However, the role of social interaction in determining the *a* and *β* parameters in the power-law and in generating a universal scaling function was unknown. Although physical space was shown to affect *β*, the individual ant’s response to a change in environment may be driven exogenously by interactions (such as encounter rate of other ants or brood items) or endogenously (some individual neural and/or physiological mechanism, operating without regard to the presence of nest-mates). Although not a focus of the present paper, we speculate that a proximate explanation for the duration–speed relationship is energetic efficiency, with a higher state of physiological arousal associated with higher movement speeds only being worthwhile if it is required for longer periods of time. Different movement periods may be associated with different forms of work of varying intensity: for instance brood care inside the nest (lower intensity) may only require short movements, but foraging outside the nest (higher intensity) may require long movements and hence may justify higher average speeds. The appropriate speed for a given duration—or given task—is likely to be set by the amount of physical space available, with a constricted environment (walls, other ants blocking the way) likely to prove an obstacle to higher speeds.

Considering the operation of the power-law 〈*v*(*T*)〉_*t*_=*aT*^*β*^, increases in *a* or *β* result in higher average speeds for all durations, but increases in *β* specifically result in a nonlinear boost to average event speeds that is particularly apparent at higher durations. This disproportionate change may result from the dynamics of social interactions, or if the movement phenomena arise endogenously it may be owing to interactions at the neuronal and/or physiological level. Given that the power-law observed inside the nest spans all observed event durations (up to about 100 s), and the underlying commonality in speed profile between short and long duration movements [7], it would seem necessary that the power-law must admit a diverse range of work tasks, both within and between different environments. These may have many brief periods of movement (such as maintaining the nest entrance) or a few long periods of movement (such as carrying food back to the colony). Therefore, adjustment to *β* would seem of first importance when constraints to the physical environment are varied to maintain the accessibility of these disparate timescales. We suggest that ants are first and foremost generalists, ‘foraging for work’ [23] when they lack a useful task, be it inside or outside the nest; as such, we expect natural selection to have equipped them with a flexible movement algorithm ready to switch between situations.

We also seek to understand better the implications of the predetermination of ant movement durations. If this is driven by social interactions, will their absence result in no discernible relationship between event duration and average speed, and a lack of universality in the speed profiles? Alternatively, if it is not dependent on social interaction, does this mean that the presence of social influences will have no effect on an ant’s behaviour? We hypothesize that the predetermination of movement durations will be a persistent phenomenon originating endogenously from some individual neural and/or physiological mechanism(s), and hence adjustments to movement durations will typically occur between movement events.

Therefore, in the present paper we have three goals. First, we track lone ants exploring outside the nest in an expansive environment lacking immediate physical constraints, to establish whether the speed–duration relationship 〈*v*(*T*)〉_*t*_=*aT*^*β*^ and universal scaling function can be identified, and if so, how they and other movement characteristics compare with the data from the inside nest regime [24]. Second, we assess the impact of social interaction by introducing it in the subtlest manner possible, by allowing chemical information such as pheromones potentially deposited by previous exploring ants to accumulate in the same arena [25–27], and compare this experimental treatment with one where the arena is always cleaned before ants embark on their survey. Third, we test whether the exploring ants are responding to social information by assessing whether the correlation between successive average event speeds is different, on average, between the two treatments. Unlike fixed aspects of the physical environment, such as the size of a colony’s nest site, pheromone deposits or cues such as cuticular hydrocarbon footprints left by previous ants are likely to be encountered in an unpredictable way by exploring ants. We anticipate that in the absence of social information, the average correlation between event speeds should be relatively higher, as in a large empty arena few adjustments to a sequence of movements should be necessary. By contrast, with socially informative chemical deposits present, ants are likely to modify their behaviour by slowly examining that region of space, for instance, and as a result we expect the correlation to be reduced. A statistically significant change in inter-event speed correlation when pheromones are present, alongside a persistent scaling function and power-law relationship, would indicate that social information affects the sequence of choices of movement events, rather than the characteristics of the movements themselves. In other words, it would indicate that, at least in some respects, response to social information (the colony level of organization) occurs intermittently and not continuously, as a result of event duration predetermination (motor planning).

## 2. Methodology and apparatus

We examine the exploratory behaviour of solitary *T. albipennis* ants in an empty experimental arena. They are small rock-dwelling ants typically no longer than 2.5 mm. Three *T. albipennis* colonies of similar sizes (approx. 150 workers and 70 brood, each with a queen) were collected from Dorset, England in March 2014 and emigrated into artificial nests. These were constructed by cutting a 50×40 mm hole out of a 75×50×1 mm piece of card, and sandwiching it between two microscope slides. The top slide had a 2 mm diameter entrance hole drilled into its centre, with a red acetate sheet underneath to darken the cavity and hence better approximate the dark rock crevices the ants inhabit in the wild.

Individual ants were automatically tracked using a digital video camera attached to a motorized gantry. An image was captured from the camera 10 times per second and used as an input to an in-house LabView^^TM^^ based program, which interfaced with the gantry motion-control system using standard ActiveX controls. The images were converted in real time from the red, green, blue output format of the camera into a hue, saturation, luminance colour space format. Following a thresholding of the luminance channel, LabView Vision Development Module image processing routines were used to record the position of the centre of mass of the single ant being tracked, as described in [28]. The program was further developed such that the physical location of the camera was dynamically adjusted via the gantry to ensure that the centre of mass remained near the centre of the image. By combining the calibrated position of the ant within the image and the real-world position of the gantry, the *x*–*y* coordinates of the moving ants were recorded every 0.1 s to an estimated accuracy of ±0.5 mm.

**Figure 1.**
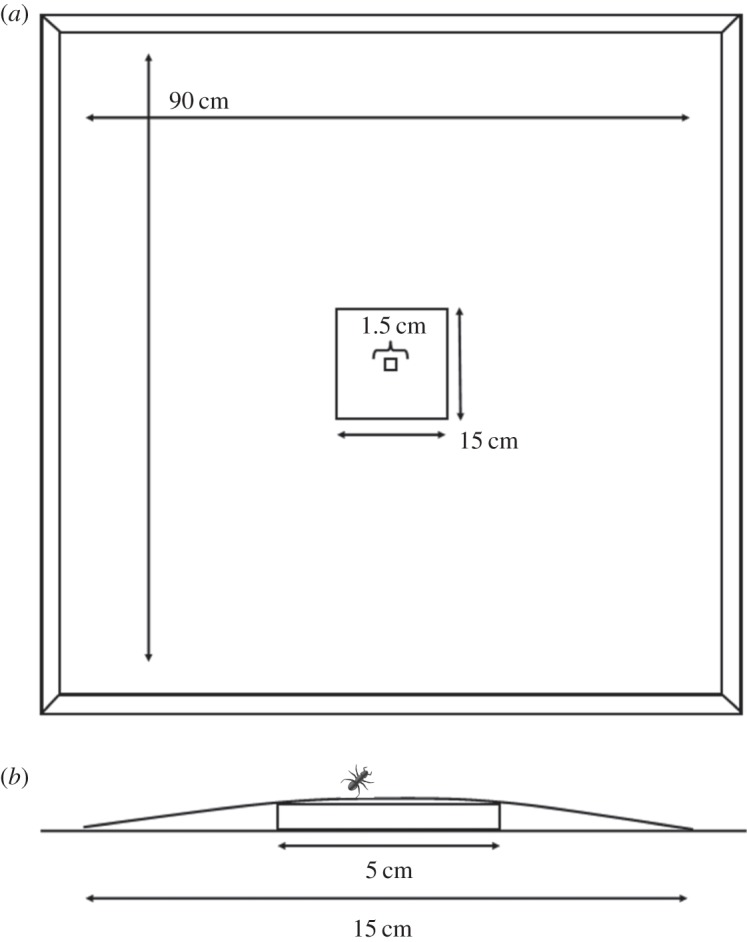
(*a*) The experimental arena. (*b*) Side view. The paper mask covers the 7.5×5 cm ant nest, and a removable 1.5 cm cover over the nest entrance allows ants to be released into the arena one by one.

The ants were subject to two experimental treatments during May 2014, where single ants explored an unfamiliar empty arena. In both treatments, a colony inside its nest was placed in the centre of an arena 90 cm square and its nest entrance covered with a 1 cm square acetate sheet to prevent ants leaving the nest. A white paper mask of 15 cm square with a 1 cm square central hole was affixed over the nest. The acetate sheet was moved aside with entomological forceps until a single ant left the nest and walked onto the mask, when the entrance was re-covered and a further 1.5 cm square white paper mask affixed to the larger mask over the entrance (figure 1). The masks created a smooth surface on the arena floor (with an elevated area over the nest), and allowed a single ant to be detected and automatically tracked without interruption. The ant explored freely within the bounds of the arena for 45 min and was then removed and not reintroduced to its colony until the end of the experiment to avoid pseudoreplication. In the first treatment (no cleaning, ‘NC’), six ants were consecutively released from the same colony on a single day and their trajectory recorded. In the second treatment (cleaning, ‘C’), six ants were also recorded but between the exploration of one ant and the release of the next the arena was cleaned to prevent chemical communication between successive ants.

The cleaning process consisted of three stages: first, an application of a weak mixture of water and detergent using a paint roller, which was removed using a window wiper; second, an application of 70% ethanol–water solution onto the arena using a test tube and paper towel; third, an application of water using a different roller and wiper. This three step process removed both the original chemical trail from the ant and also any trace of the cleaning products.

Alternating between treatments and colonies, 45 min trajectories for a total of 18 ants in each of the two treatments were recorded over six consecutive days, 12 ants from each of the three colonies. As in the no cleaning treatment the first ant explores a clean arena, and hence its behaviour would be expected to be similar to ants in the cleaning treatment, we exclude the first ant of the sequence of six. This results in a dataset of 15 ants in the no cleaning treatment and 18 ants in the cleaning treatment. The ensuing analysis is generally presented by colony.

## 3. Data processing and analysis

We denote the three experimental colonies as *C*^1^, *C*^2^ and *C*^3^. We add a subscript NC for the no cleaning treatment and a subscript C for the cleaning treatment, for instance *C*^1^_NC_ and $C_{C}^{1}$. Otherwise, we use the same notation previously established in Christensen *et al.* [7]. When we analyse data from the experimental treatments in the previous study of ants moving inside the nest [24], the two nest cavity sizes of 35×28 and 55×44 mm are referred to as the smaller and larger nest.

We sought to reduce the influence of small-scale fluctuations in the data by employing a coarse-graining technique, which also enhances comparability with previous research [7]. As the data has similar characteristics (data points are spaced by just over 0.1 s), the data presented here uses a coarse-graining factor λ=8 such that eight data points are smoothed to obtain an average unit time interval of 0.83 s.

**Figure 2.**
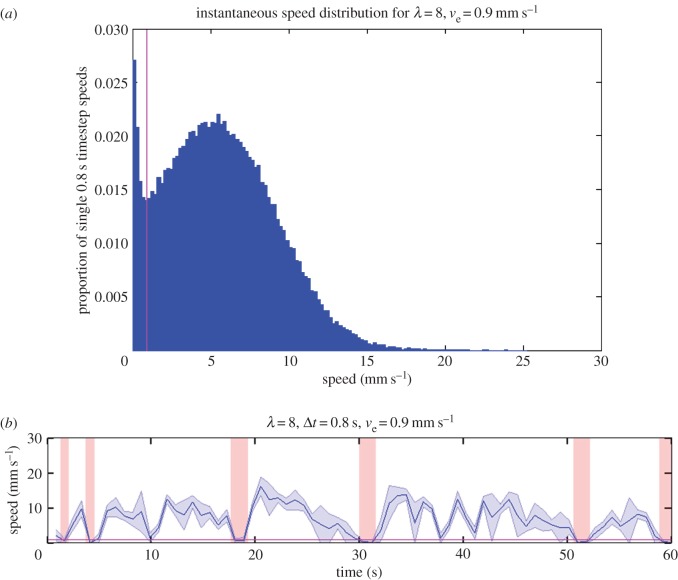
(*a*) The speed distribution of all ants at λ=8. The empirically determined activity event speed threshold *v*_*e*_ is shown as the vertical magenta line, at 0.9 mm s^−1^. (*b*) Illustration of the event definition process. A 60 s period of exploration is shown for λ=8. Estimated error bars in speed are shown as a blue band around the mean. To define the end of a period of activity, the speed must be less than or equal to a threshold of 0.9 mm s^−1^ for a single time step or longer. The speed threshold is shown as the horizontal magenta line, while bands of red on the *y*-axis indicate a stopping period according to this criterion. Higher λ leads to tighter error bars and hence a lower speed threshold because one-step displacements are larger, leading to reduced fractional error in the speed with a fixed tracking error.

It is not possible to define an activity event as a sequence of non-zero speeds bounded by zero speeds because of two factors: first, a small degree of tracking error in the automated system results in small non-zero speeds and second, the near-continuous movement of the ants. By contrast, the previous study [7] used manual tracking so that stopping periods could be recorded as zero speed. Hence, we define an activity event by reference to a threshold speed *v*_*e*_, below which we say the ant is inactive. The distribution of instantaneous speeds for all 33 ants when the coarse-graining factor λ=8 is shown in figure 2*a*. Bins of width 0.2 mm s^−1^ are used to construct the histogram. A bimodality is observed with the first peak near to zero. The speed threshold for an event end is determined empirically, by reference to the turning point in the bimodal speed distribution, which we take to be the cut-off in discernibility between non-zero and zero speeds. As the distribution shown in figure 2*a* has peaks at [0, 0.2) mm s^−1^ and [5.4, 5.6) mm s^−1^, while the low point between the peaks is at [0.8, 1.0) mm s^−1^, the speed threshold for an activity event end is therefore chosen to be *v*_*e*_=0.9 mm s^−1^ for λ=8. Figure 2*b* illustrates the spacing of events using this speed threshold as a sequence of red shaded areas where the ant is said to be (often momentarily) inactive. Error bars are estimated according to $\sqrt{{\left(0.5/\Delta x\right)}^{2}+{\left(0.5/\Delta y\right)}^{2}}$, where Δ*x* and Δ*y* are one-step displacements, and 0.5 mm is the fixed error in the *x*- and *y*-coordinates, based on an estimate that the gantry can track to the nearest millimetre in both dimensions. This threshold shifts according to the coarse-graining factor λ employed: the electronic supplementary material contains further graphs and data for λ=2,4,16 in addition to λ=8 (electronic supplementary material, figure S1).

For the analysis of the relationship between average event speed and event duration, as the observed ant activity durations span over two orders of magnitude, from 0.8 s to over 200 s, a log-binning process (as described in [29]) is employed to improve the accuracy of average speed estimates for longer duration events, by capturing more infrequent long duration events in a single bin. Fitting to logarithmically binned data allows each part of the distribution to contribute equally to the fit, leading to a less biased power-law exponent estimate [30]. The process sets bin limits according to [*a*^*j*^*a*^*j*+1^), where *a* is a number slightly more than 1 and *j* are a sequence of integers, and it produces exponentially increasing bin sizes. We chose *a*=1.2 to obtain adequately sized bins in the relevant range (electronic supplementary material, table S7 repeats analysis for *a*=1.1 with qualitatively the same results).

The first *j* in the sequence is chosen such that the first bin is slightly larger than the minimum width suggested by λ. With the coarse-graining factor λ=8 and the original trajectories having Δ*t*^(1)^=0.1±0.004 s, the shorter events are clustered around multiples of Δ*t*^(8)^=0.8 s. Fixed-width bin limits centred on multiples of 0.8 s are therefore used up to the first bin created with the log-bin method, the smallest possible with a width ≥0.8 s, which has its lower limit slightly adjusted to match up with the last fixed-width bin. Events falling within the bins set according to the method described above are averaged on both the time and speed dimension, rather than setting their time equal to the bin centre, so that less information is lost.

We ran general linear mixed models (GLMM) to assess whether the average speed of events, the average event duration and the average stopping duration between events was significantly different between treatments. The predictors were treatment (fixed effect) and colony number (random effect). While the average event speed was approximately normally distributed, the event duration and stopping duration were not, and so they were log-transformed before analysis. As a result, geometric rather than arithmetic means were obtained. We also used a GLMM to assess whether the cleaning and no cleaning treatments had an effect on the speed–duration power-law relationship. The response variable was log_10_(average event speed) and the predictors log_10_ (event duration) as a covariate, treatment as a fixed effect and the interaction between the two, and with colony as a random factor.

**Figure 3.**
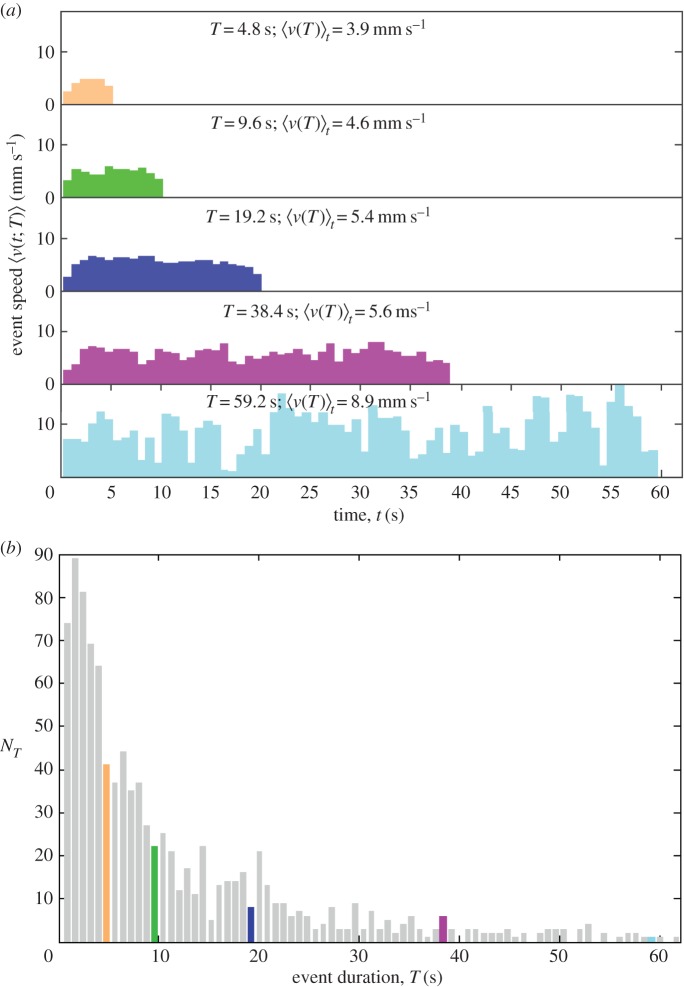
(*a*) Examples of average event speed profiles 〈*v*(*t*;*T*)〉: speed versus time *t* averaged over all events with duration *T* for the six ants in $C_{C}^{2}$. The event speed profiles have durations *T*=4.8 s (orange), 9.6 s (green), 19.2 s (blue), 38.4 s (magenta) and 59.2 s (cyan), and the number of events *N*_*T*_ is 41, 22, 8, 6 and 1, respectively. In general, the longer the event *T*, the higher the average event speed for the particular profile 〈*v*(*T*)〉_*t*_. (*b*) The number of events *N*_*T*_ versus event duration *T* for $C_{C}^{2}$. The trend is for the number of events to decrease with event duration, which is binned into units of 0.8 s. The highlighted events are those displayed in (*a*). The total number of events shown $\sum_{T}N_{T}=1024$.

If the average speed–duration profile for all events of a particular duration *T*, of the sort illustrated in figure 3*a*, is rescaled by its average speed, its average speed is 1. If each point in time *t* is divided through by total duration *T* it lies in the region [0, 1]. This normalization allows speed–duration profiles of different durations to be compared, and their essential characteristics to be condensed into one curve. When 〈*v*(*t*;*T*)〉/〈*v*(*T*)〉_*t*_ is plotted against *t*/*T*, or in other words an average speed–duration profile is rescaled by the average speed for that profile, and time on the *x*-axis is rescaled by the movement duration, and this is repeated on the same axes for the whole duration range of observed profiles, the data collapse and indicate the scaling function $G(t/T)=\langle v(t;T)\rangle /{\left\langle v(T)\right\rangle }_{t}$, as described in Christensen *et al.* [7]. Because these profiles range in duration from 0.8 to 123.4 s, when they are rescaled by *T* they have differing numbers of points in the region [0, 1]. Therefore, to obtain an average of numerous profile curves, linear interpolation is used to obtain the profile speed at *t*/*T* at set intervals of 0.02 between 0 and 1 for each profile, before averaging these interpolated values.

We assessed whether the correlation between successive average event speeds is different, on average, between the two treatments. As a preliminary step in calculating an average event speed correlation for each colony and treatment, we tested whether the correlation coefficient for each ant from the same colony and treatment was sufficiently similar to justify combining them together into a single estimate for that colony. Deviations of individual correlations from the proposed weighted average are summed in the calculation of a *χ*^2^-test statistic, which indicates whether there is unexpected variance given an assumption of normally distributed observations around the same underlying mean. If the null hypothesis that the correlations are the same is not rejected, we can proceed to calculate a weighted average correlation coefficient [31]. If the correlations are found to be statistically different, there is further deliberation before continuing. Before comparing coefficients we use the Fisher *r* to *z* transformation, which converts Pearson’s *r* into a normal distribution between ±∞ with a stabilized variance [32]. The *χ*^2^-value for the transformed *z*-coefficients is calculated according to $\chi^{2}=\sum_{i}(n_{i}-3){\left(z_{i}-z_{w}\right)}^{2}$, where *z*_w_ is the weighted value. The critical values are $\chi_{4,0.05(2)}^{2}=9.488$ for the no cleaning treatment (d.f.=5−1=4) and $\chi_{5,0.05(2)}^{2}=11.070$ for the cleaning treatment (d.f.=6−1=5). The calculated *χ*^2^ values are above the critical values for $C_{C}^{1}$ and $C_{C}^{3}$, but because of sources of inter-individual variation such as physical maturity and experience, and indeed, intra-individual variability [17], it is perhaps unrealistic to expect that the *z*-value should be the same for the five (no cleaning treatment) or six (cleaning treatment) ants from each colony. Therefore, we justify the calculation of a common or weighted *z*_w_ for $C_{C}^{1}$ and $C_{C}^{3}$ by noting that only one *z* is below the mean in each case, which suggests that any bias in *z*_w_ is in a downward direction, acting against the finding of statistical significance in this case, where we observe the cleaning treatment to generally have a higher correlation. The weighted *z*_w_ is calculated according to $z_{w}=\sum_{i=1}^{k}(n_{i}-3)z_{i}/\sum_{i=1}^{k}(n_{i}-3$), where *n*_*i*_ is the number of pairs of successive average event speeds being correlated, which is one fewer than the total number of events for each ant. The *z*_w_ for each treatment is then compared by calculating a *Z*-score via $(Z=z_{w(\mathrm{NC})}-z_{w(C)})/(\sqrt{1/(n_{1}-3)+1/(n_{2}-3)}$), where *n*_1_ and *n*_2_ are calculated according to $\sum_{i=1}^{k}(n_{i}-3$), where *k* is 5 or 6 ants for NC or C, respectively. The critical value is *Z*_0.05(2)_=1.960. The standard error of *z*_w_ is approximated by $\sigma_{z_{w}}=\sqrt{1/(n-3)}$, where *n* is calculated as just described. To calculate the weighted correlation for each treatment *r*_w_ as shown in figure 9, the respective *z*_w_ for each colony was combined according to the *z*_w_ formula, where the *n*_*i*_ are the number of events recorded for each colony. The reverse *r*-to-*z* transformation was used to obtain *r*_w_ and *σ*_*z*_r__ values for graphs.

General linear mixed models were run in SPSS^r^ v.21, while all other data analysis was performed in MatLab^r^ R2014a.

## 4. Results

### 4.1 Differences in averages between movements within the nest and outside the nest

**Figure 4.**
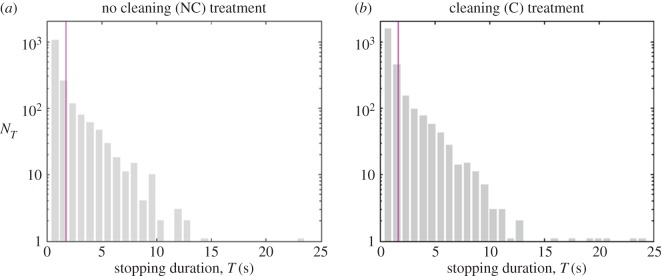
The distribution of stopping durations for the (*a*) no cleaning and (*b*) cleaning treatments (all colonies). The arithmetic mean stopping duration is indicated by a magenta line; the geometric mean is not significantly different between treatments (figure 5*b*; electronic supplementary material, table S3). Note that there are 15 ants in the no cleaning treatment and 18 in the cleaning treatment.

The relationship between longer event duration and higher average speed is qualitatively the same as that observed inside the nest [7] (figure 3), hence the ants sometimes reach very low instantaneous speeds (near the event speed threshold) during a movement event, confirming that an ant does not necessarily stop after substantially reducing its speed (figures 2*b* and 3*a*, especially the average speed profile for events of 59.2 s). Longer events are progressively less frequent (figure 3*b*). Concerning periods of inactivity, there is also a rapid decrease in the frequency of longer stopping durations (i.e. time spent below the speed threshold): most stops are for one time step (figure 4). This is similar for both treatments.

**Figure 5.**
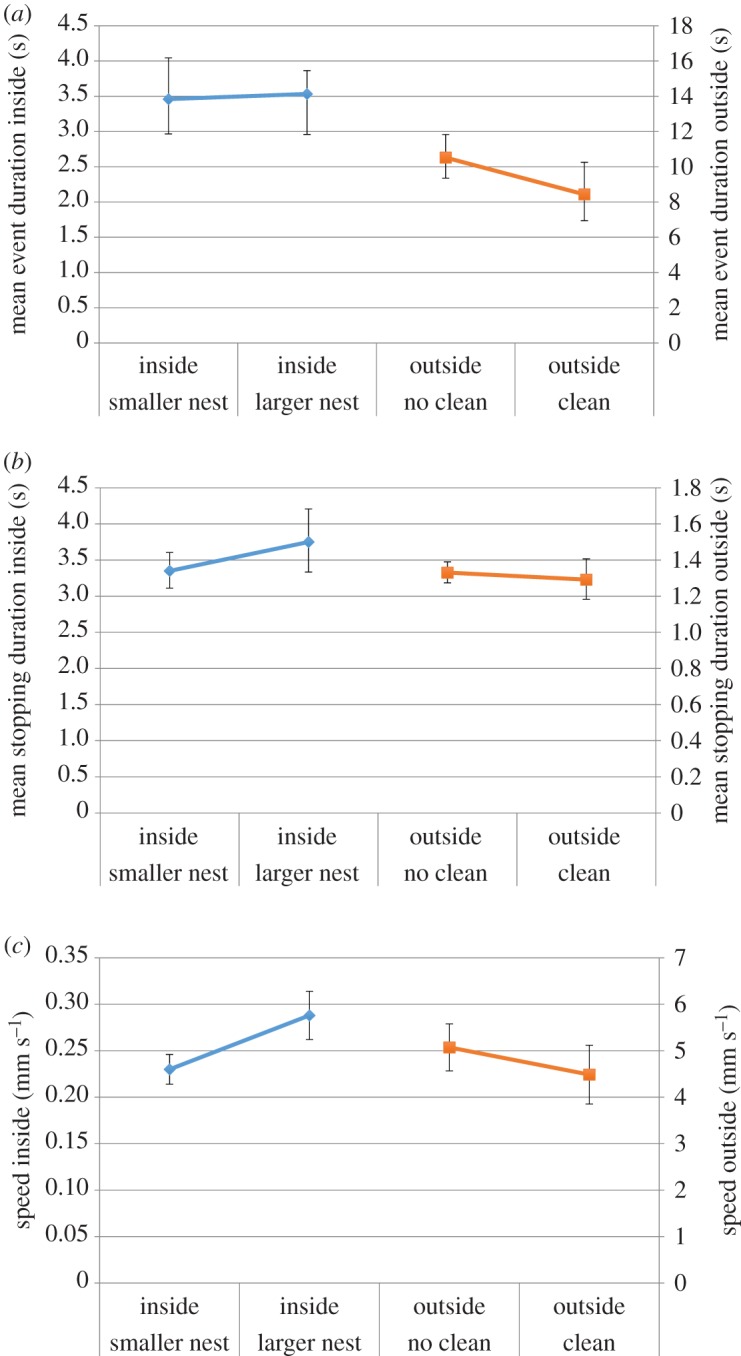
95% confidence intervals (CIs) of the mean shown, two vertical axes are employed. (*a*) Outside the nest, the geometric mean event duration is significantly longer in the no cleaning treatment. Inside the nest, it is not significantly different between nest sizes. (*b*) Outside the nest, the geometric mean stopping duration is not significantly different between cleaning and no cleaning treatments. Inside the nest, ants stop moving for longer in the larger nest. (*c*) Outside the nest, the average event speed is significantly higher in the no cleaning treatment. Inside the nest, the average event speed is significantly higher in the larger nest.

While the (geometric) mean event duration is 3.53 s in the large nest and 3.46 s in the small nest (not significantly different, *n*=16 480, *t*=1.733, *p*=0.083), average event duration outside the nest is considerably longer. In the no cleaning treatment, it is 10.52 s, and 8.43 s in the cleaning treatment (*n*=4289, *t*=−5.580, *p*<0.001); the shorter time in the cleaning treatment may result from caution when ants cannot detect their nest-mates (figure 5*a*; electronic supplementary material, table S2). This explanation may be tested with more data in future research by examining whether the average speed in the no cleaning treatment rises with successive exploring ants above a constant average speed in the cleaning treatment. The (geometric) mean stopping duration is not significantly different between treatments: 1.33 s in the no cleaning treatment and 1.29 s in the cleaning treatment (*n*=4256, *t*=−1.458, *p*=0.145). Inside the nest, however, the stopping duration is longer in the larger nest, 3.75 s compared with 3.35 s in the smaller nest (*n*=16 421, *t*=5.105, *p*<0.001, see figure 5*b*; electronic supplementary material, table S3).

Examining event speeds further, the average event speed is calculated to be significantly lower in the cleaning treatment $\bar{v_{\mathrm{NC}}}=5.07\;\mathrm{mm}\;s^{-1},\bar{v_{C}}=4.49\;\mathrm{mm}\;s^{-1}$ (*n*=4289,*t*=−9.052, *p*<0.001). We again suggest that the lower speed may be attributable to caution in unfamiliar environments. As one might expect, the speed of ants exploring outside the nest is considerably higher than for those moving among their nest-mates inside the nest. The mean event speed in the larger nest is significantly higher than in the smaller one $\bar{v_{\mathrm{large}}}=0.29\;\mathrm{mm}\;s^{-1}$, $\bar{v_{\mathrm{small}}}=0.23\;\mathrm{mm}\;s^{-1}$ (*n*=16 480,*t*=12.275, *p*<0.001, see figure 5*c*; electronic supplementary material, table S4).

Colony identity did not have a significant effect in any of the GLMMs (electronic supplementary material, tables S1–S4).

### 4.2 Power-law, scaling function and successive event speed correlations

**Figure 6.**
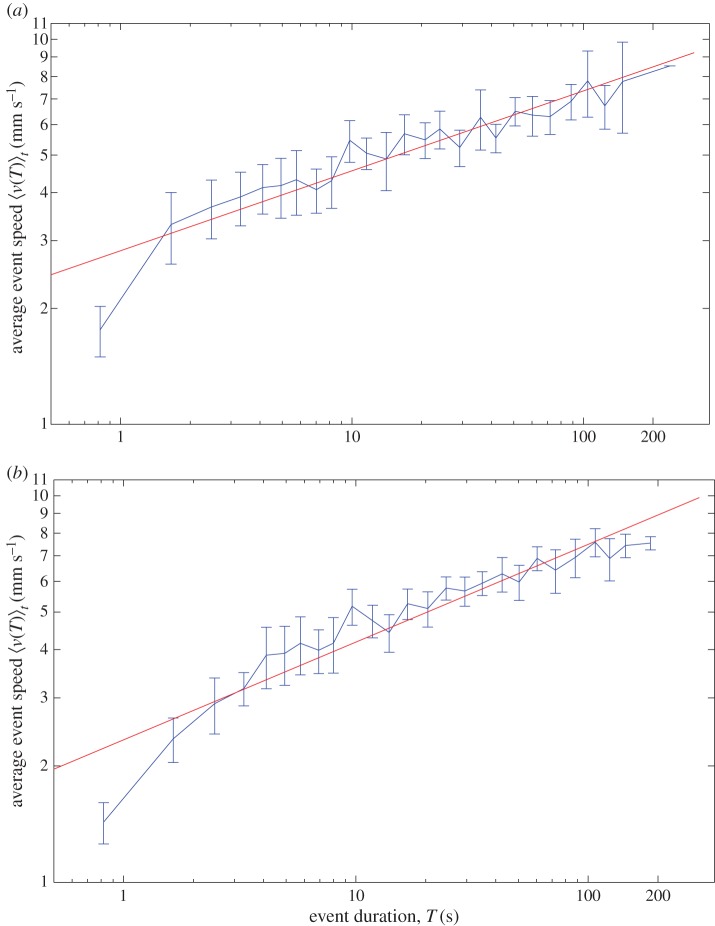
Average event speed 〈*v*(*T*)〉_*t*_ versus event duration *T* for (*a*) no cleaning treatment and (*b*) cleaning treatment. Data for *C*^1^. Error bars indicate 95% CI around the mean. Data points without an error bar originate from one event only. The red line on the log–log plot indicates a power-law relationship 〈*v*(*T*)_*t*_〉=*aT*^*β*^. Across all colonies, *β*_NC_=0.22±0.02,*β*_*C*_=0.24±0.03,*a*_NC_=2.82± 0.27 and *a*_*C*_=2.45±0.27, indicating a sub-linear increase in average speed with the duration of an event. There is a departure from trend for very short and very long events, which we suggest is attributable to insufficient acceleration time and a plateauing effect at an individual ant’s maximum speed.

We find a linear relationship between event duration and average event speed on log–log axes, indicating an approximate power-law relationship 〈*v*(*T*)〉_*t*_=*aT*^*β*^ over ≈1.5 orders of magnitude variation of time. Figure 6 shows the relationship for *C*^1^ in both treatments. There is a departure from trend in the first data point at *T*=[0.4,1.2) s, and also at the longest duration events, with the average speeds being lower than those suggested by the fitted *a* and *β*. We suggest that this results from insufficient time for acceleration in the short interval, and to a plateauing effect when individual ants approach their maximum speed, respectively. The graphs for all colonies are available in the electronic supplementary material, figure S2.

**Figure 7.**
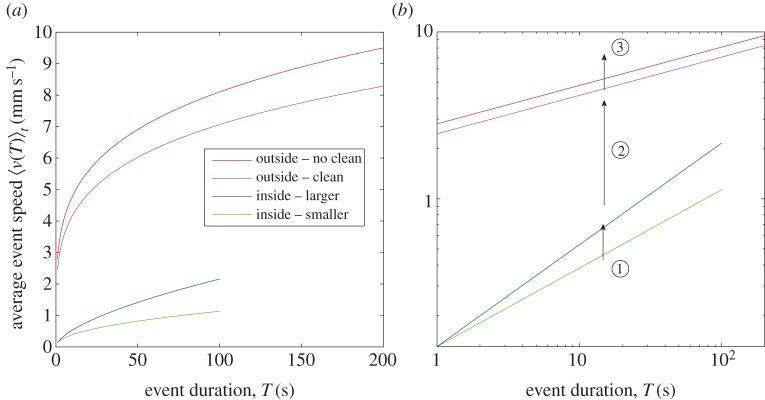
(*a*) The power-law relation 〈*v*(*T*)_*t*_〉=*aT*^*β*^ shown for the average exponents across the four treatments studied with a representative range of durations. Log scale shown on axes in (*b*). (1) Inside the nest, nest dimensions enlarged—*β* changes (increases), *a* constant. (2) Shift from inside the nest to outside the nest—*β* changes (decreases), *a* changes (increases). (3) Remaining outside the nest, social information introduced—*β* unchanged, *a* changes (increases).

Over all colonies, the exponent *β* is not significantly different between the no cleaning and cleaning treatments (*β*_NC_=0.22, *β*_*C*_=0.24, *t*=1.388, *n*=154, *p*=0.167). However, the intercept is significantly lower in the cleaning treatment (*a*_NC_=2.82, *a*_*C*_=2.45,*t*=−2.609,*n*=154, *p*=0.010), indicating that for a given duration, the ants are, on average, moving at a lower speed when they cannot detect the presence of previous nest-mates. This finding is repeated across coarse-graining levels (see the electronic supplementary material, table S1; significance is only not found for λ=16). The similarity in exponent *β* between treatments indicates that low-level variation in external social environment is insufficient to drive the sort of nonlinear relative increase in speed at longer event durations, as was seen when the size of the physical environment was manipulated in the inside nest study [7]. The parameters *a* and *β* outside the nest (in both treatments and all colonies) are significantly different compared with those found inside the nest (95% CIs do not overlap). The coefficient *a* outside is significantly higher (*a*_NC_=2.82±0.27,*a*_*C*_=2.45±0.27, and the average *a* inside the smaller nest =0.12±0.01, inside the larger nest =0.12±0.03), while the exponent *β* is significantly lower (*β*_NC_=0.22±0.02, *β*_*C*_=0.24±0.03, and the average *β* inside the smaller (35×28 mm) nest =0.47±0.06 and inside the larger (55×44 mm) nest =0.60 ±0.07; 95% CIs given, see also table 1 and [7]). This finding is consistent with our hypothesis that *β* is principally determined by the size of the physical environment. The comparison with the inside the nest exponents is summarized in figure 7.

**Table 1.** The estimate and 95% CIs of the exponents *β* and coefficients *a* (in units of mm s^−1^) associated with the power-law relationship 〈*v*(*T*)〉_*t*_=*aT*^*β*^ for the three investigated colonies in each of the two treatments, no cleaning (NC) and cleaning (C). (The values are based on a linear regression fitted to the log–log relationship between average event speed and event duration.)

colony	*C*^1^_NC_	*C*^1^_C_	*C*^2^_NC_	$C_{C}^{2}$	*C*^3^_NC_	$C_{C}^{3}$
exponent *β*	0.21	0.25	0.22	0.26	0.23	0.22
95% CI for *β*	±0.03	±0.04	±0.04	±0.04	±0.05	±0.04
coefficient *a*	2.8	2.3	2.6	2.3	3.1	2.8
95% CI for *a*	±0.3	±0.3	±0.3	±0.3	±0.4	±0.4

**Figure 8.**
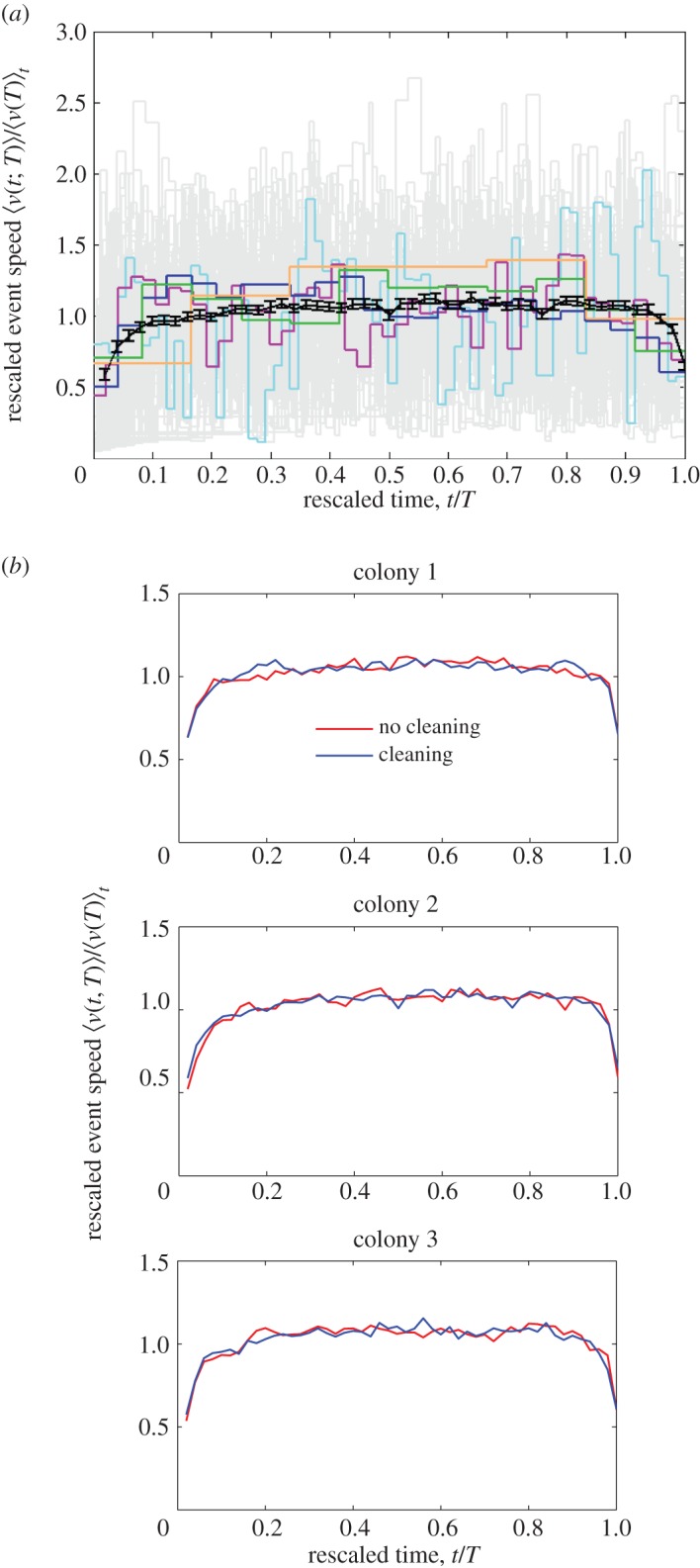
(*a*) Rescaled event speed 〈*v*(*t*;*T*)〉/〈*v*(*T*)〉_*t*_ versus rescaled time *t*/*T*. Data for colony $C_{C}^{2}$. Rescaling the speeds in an event of duration *T* with its average speed aligns the event speed profiles. Grey lines represent the rescaled event speed profiles for all the $\sum_{T}N_{T}=1024$ events. The highlighted events indicated with orange, green, blue, magenta and cyan are the same as in figure 3. The average rescaled event speed profile (black curve) indicates the scaling function G. Error bars show 1 s.e.m. (*b*) Scaling function G for the three colonies in both no cleaning (red) and cleaning (blue) treatments. After a period of acceleration the average speed is reached for most of the event, followed by a shorter period of deceleration at the end. This patterns holds in both treatments.

As noted, there is a higher variation in speeds comparing outside the nest with inside it, as one might expect. However, there is an underlying similarity and hence predictability in the event speed profiles. This suggests similarity in the neural and/or physiological mechanism(s) determining movement characteristics across ants and colonies. As described in the Data processing and analysis section, if all the profiles of different duration *T* are rescaled by their average speed and total duration, a so-called data collapse is obtained which indicates a single scaling function G [7]. An example is shown as the black curve in figure 8*a*, which is obtained by further averaging across all the rescaled profiles for different durations, which number 95 in the case of $C_{C}^{2}$. This function initially increases up to around *t*/*T*≈0.10 where it attains a constant ≈ 1 before decreasing at *t*/*T*≈0.95 (figure 8*b*); whereas for ants tracked inside the nest these points are *t*/*T*≈0.05 and *t*/*T*≈0.90, respectively [7], which means that, on average, ant movements outside the nest initially accelerate for longer and decelerate more rapidly towards completion. This can be understood by considering that much higher average speeds can be reached outside the nest and hence longer periods of acceleration are required. Faster deceleration may be characteristic of exploratory activity where an ant is rapidly changing direction, in contrast with inside the nest where it may be approaching a nest-mate. The shape of the scaling function is further indication that causation proceeds from event duration to average speed, as the long period of deceleration that might accompany a high speed, high momentum, long duration chain of causation is even less in evidence than for the curve associated with movement inside the nest.

For each of the three colonies the correlation between successive average event speeds was higher in the cleaning treatment than in the no cleaning treatment (table 2; only not significant for λ=16, see the electronic supplementary material, table S5). Considering that most stopping durations are of one time step in duration for λ=8 (figure 4), or around 0.8 s, we suggest that λ=16 is an excessive degree of coarse-graining, as it results in time steps of around 1.6 s, in comparison to the original data recorded with $\Delta t^{\left(1\right)}=0.1\pm 0.004\;s$. As a result, the boundaries of many activity events (periods of stopping) may be averaged out and lost in the analysis for λ=16, and hence we consider that the results for λ=2,4,8 are more reliable.

**Table 2.** Correlation coefficient for consecutive average event speeds in a colony/treatment (five ants in NC, six in C). (Correlation coefficients are converted into *z*-scores using Fisher’s *r*-to-*z* transformation, which are combined into a weighted *z*_w_ for each treatment that is then compared to assess the significance of the difference between the correlations in the two treatments. The reverse transformation is used to provide a weighted *r*_w_. See the Data processing and analysis section for more details.)

colony	*C*^1^_NC_	*C*^1^_C_	*C*^2^_NC_	$C_{C}^{2}$	*C*^3^_NC_	$C_{C}^{3}$
*zχ*^2^ (critical value)	8.3 (9.5)	28.1 (11.1)	2.7 (9.5)	8.1 (11.1)	4.2 (9.4)	11.2 (11.1)
weighted *z*_w_	0.34	0.54	0.27	0.45	0.15	0.30
*Z*-score		−3.43		−3.50		−2.89
*p*-value (two-tailed)		0.0006		0.0005		0.004
*r*_w_	0.33	0.49	0.27	0.42	0.15	0.29

**Figure 9.**
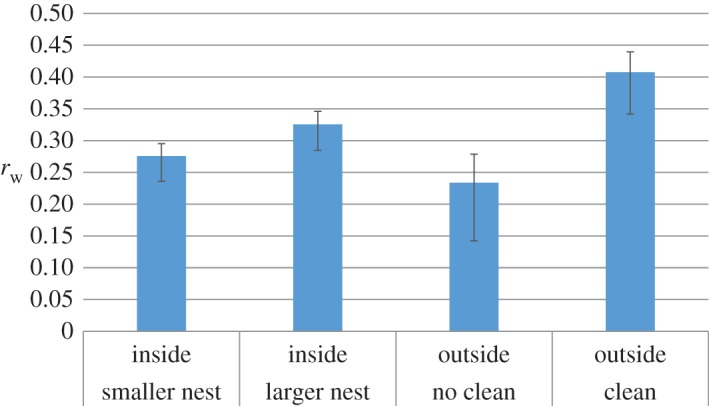
Weighted correlation *r*_w_ between successive average event speeds, inside and outside the nest, for different treatments. The outside, cleaning treatment correlation is the highest, when individual ants encounter no social information (physical or chemical) from other ants. 95% CIs shown. See also the electronic supplementary material, table S6.

A weighted average correlation across colonies is obtained for each treatment, both inside and outside the nest (λ=8). The cleaning treatment is found to have a significantly higher correlation than both the no cleaning treatment, and the event speed correlations found inside the nest (figure 9; electronic supplementary material, table S6), which we interpret as resulting from the lack of social information being integrated into the ants’ exploratory movement process. The inside, larger nest treatment has a significantly higher *z*_w_ than the inside, smaller nest treatment, which again may be owing to less intensive social interaction.

The outside, no cleaning treatment *z*_w_ is not significantly different from the inside, smaller nest treatment, but it is significantly lower than the inside, larger nest treatment. While we might anticipate a steady increase in correlation in figure 9 from left to right, as social interaction progressively decreases, this departure from trend may be explained by a switch from physical (touch-based) interaction between ants to chemical (social pheromone-based or cue-based) interaction. In this case, the presence of pheromones may have more impact on exploratory movement than the density of nest-mates and brood items has on the movement of an ant looking for useful jobs to perform. This may be because such pheromones are signals, which means they have evolved specifically to influence the behaviour of the receiver; ants are known to employ both attractive [33] and repellent signals for their nest-mates [34]. It is also possible that cues such as cuticular hydrocarbon footprints are left by previous exploring ants in the no cleaning treatment, which may alternatively contribute in whole or in part to the observed change in movement behaviour. Whether or not pheromones are purposefully deposited, for instance with the intention of marking explored empty space [34], it would seem that prior ants have a social influence on their succeeding nest-mates.

## 5. Discussion

We find that the speed–duration relationship 〈*v*(*T*)〉_*t*_=*aT*^*β*^ is retained outside the nest across the full range of ant movement speeds. Furthermore, it is retained across different regimes of social interactions, from our no cleaning treatment where the interaction is slight, to a small cavity with more than 100 ants and 50 brood items, where the interaction is intensive [7]. It is also retained across the multiple colonies examined, three in this study and three in the study inside the nest [7], despite possible variation with respect to different colony-level behavioural syndromes, for instance, which include factors like collective caution and aggression [35,36]. The power-law parameters *a* and *β* are significantly different to inside the nest (higher and lower, respectively). The introduction of social interactions via chemical deposits does not change *β*; it does slightly increase *a*. The effect on the parameters of the four different experimental treatments is summarized in figure 7. We suggest that *β* can be understood as indicating the range in average movement event speeds associated with the work tasks available. Tasks outside the nest may be closer together in speed, albeit generally faster from the greater physical space (1–9 mm s^−1^, a 9× variation, figure 6), and hence a lower *β* is needed to switch between them along with a higher *a*. On the inside there is a broader range in work task speeds, from the very slow to the moderately fast (0.1–2 mm s^−1^, a 20× variation, [7]), hence a higher *β* is required along with a lower *a*.

While there is much variation in speeds in comparison between inside and outside the nest, as one might expect, only small differences are noted between the underlying movement regimes, by comparing the scaling function G. Outside the nest this function initially increases up to approximately *t*/*T*≈0.10, where it attains a constant ≈1 before decreasing at *t*/*T*≈0.95 (figure 8*b*); whereas for ants tracked inside the nest these points are *t*/*T*≈0.05 and *t*/*T*≈0.90. Within-experiment treatments (physical environment changing from small to large nest; social environment shifting from absent to present) did not markedly change the location of these turning points. It seems possible therefore that two distinguishable but closely related behavioural regimes are in operation: one for the ants in their nest and one for exploration outside. For an experimenter trying to characterize the behaviour of individual agents in a social group, this suggests that a broad range of data from differing social and genetic environments could be relevant to the task of prediction and control within such loosely defined regimes. It remains to be seen whether introducing multiple ants to an arena so they can physically interact, for instance through antennation, would result in the indication of a further distinguishable scaling function G, but an identical method to that detailed in this and previous research [7] could establish this. Indeed, the more the ant complex system is studied in different physical and social environments, the more apparent the simple underlying movement rules should become.

One might expect the combination of simplicity and scalability implied by the enduring power-laws and scaling functions in the behaviour of an ant, given an orientation towards servicing its colony in whatever way contingency demands. Rather than patterns of ant activity being specialized to particular environments, and given the weak temporal structure in task allocation observed in similar species of ant [37,38], we suggest that the data presented here are further evidence for an emergent division of labour in ant societies, rather than a physically determined one. In other words, mature worker ants may be generalists who are equally as adapted to ‘foraging for work’ [39] inside the nest where shorter movements are typical (finding detritus to clear, walls to mend, brood to tend) as they are to foraging for resources outside the nest where longer duration movements are required (to find new sources of food or new potential nest sites). In this conception of task allocation, an individual ant’s decision whether to engage in a task depends on whether it is in a location where execution of that task benefits the colony [23]. A study of the Florida carpenter ant *Camponotus floridanus* found that, while younger workers are indeed biased towards choosing within-nest tasks, older workers are flexible in their task choice behaviour [40]. A further study of *T. albipennis* found that individual efficiency is not predicted by how specialized workers were on the respective task, and indeed that worker allocation to tasks was unrelated to their ability to perform them [41]. Therefore, given the apparent similarity shown here between activity events inside and outside the nest, a parsimonious explanation of what the worker ant is doing would seem to be that it is a roving interchangeable part, moving with the goal of putting itself somewhere useful to the colony. That is not to say that there is no inter-individual variation in capability—our analysis is at the level of the colony—but as far as movement is concerned, each ant seems to be equipped to tackle tasks with the same flexible movement procedure.

Our results indicate that the duration of an activity event is determined before commencement in ants exploring outside their nest. This predetermination is inferred by considering the following: (i) the universal speed profile for events showing ants travelling at a constant speed, on average, for most of a movement; (ii) the low instantaneous speeds often reached during long events with high average speeds, which seems to rule out the possibility of higher average speeds causing longer events; and (iii) the observed sub-linear power-law relationship between the duration of a movement and its average speed, which indicates the existence of a persistent internal mechanism connecting these two variables. We also recall the previous findings of research on the same ant species inside its home nest showing longer typical event durations for a given average speed in smaller nest sites [7]. We further infer that duration predetermination is not dependent on social interactions, but apparently arises endogenously from the neural and/or physiological processes at work in individual ants, as there is no interaction between ants in our cleaning treatment, and yet the power-law relationship and similar scaling functions are still found.

A significant change in behaviour occurs when chemical deposits (pheromones and/or cues) are introduced to the arena by previous exploring ants [25–27]. The correlation between the average speeds of successive events is lower, as shown in table 2. In the absence of social information, few adjustments to a sequence of exploratory movements should be necessary in a large empty arena. By contrast, with chemical deposits present, ants are likely to modify their behaviour by changing direction or searching the area, reducing the correlation between event speeds as a result. Considered together with the scaling functions and duration–speed regressions, this change in average correlation suggests that the ants do respond to social cues, but in a way which does not interrupt their characteristic movement pattern (the maintenance of a constant average speed for the duration of the movement, with an initial 10% acceleration period and a final 5% deceleration period). Instead, these findings suggest that the change in movement durations occurs *between* events.

When we consider that previous research [7] has shown the robustness of the scaling function under intensive social interaction (66–120 other ants were present in the same small nest cavity), we suggest that only changing movement durations between events may be an adaptive feature of ant brain function. Rather than engage in locomotion and decision-making at the same time, it may be less cognitively demanding to plan movements sequentially. Certain relevant social information may be collected during a movement, and processed when at rest, or alternatively we suggest that it would be simpler for the ant to both collect and process the information when its current movement is complete. Intermittent responsivity to at least some aspects of the social environment during movement may be a characteristic present in other taxa. A fascinating example of another intermittent perception-action system at a different level of biological organization is the human visual system and saccadic eye movements when looking at objects. Detailed visual inspection depends on receiving information during fixation pauses between a sequence of saccades as the brain seeks out task-relevant information; when eyes are moving rapidly no useful information is collected [42]. Eye saccades’ movement characteristics are determined before commencement [42]. There is a consistent nonlinear relationship between the magnitude of a movement and its duration and also its maximum velocity [43], known as the main sequence [44]. This type of intermittent control, associated with movement between perceptual updates, may be favoured where the quality of perceptual information is degraded during motor activity.

Significant deviation of eye saccades from the main sequence is regarded as a sign of pathology [45]. If the speed–duration relationship is found to be present in other taxa, assessment of a particular animal or group’s behaviour can be made graphically as in figure 6, after a long enough period of suitable video tracking. When compared with a well-established empirical standard for a particular social and physical environment, it may similarly be used as an indicator or early warning sign of higher-level malfunction. It may be especially useful for the monitoring of specific animals of concern in a group, because as demonstrated in previous research [7], the relationship can persist despite intensive social interaction. Other such markers of health impairment have been identified. For instance, the complexity of behavioural sequences can be used to evaluate stress conditions in chickens [46] and dolphins [47]; fractal long-range correlations of behavioural sequences have been used to identify sick chimpanzees in the wild [48]; and fractal analysis of human gait rhythm can be used to assess ageing and chronic disease [49]. The speed–duration relationship could be added to this repertoire of metrics that can be obtained through non-invasive surveillance for the purpose of monitoring animal welfare.

## 6. Conclusion

In our study of individual ants moving outside the nest we found in both treatments (cleaning and no cleaning), a data collapse of event profiles onto a single scaling function can be obtained, and the average speed–event duration relationship 〈*v*(*T*)〉_*t*_=*aT*^*β*^ again holds, with *a* and *β* significantly higher and lower than inside the nest, respectively. Analysis of the correlation between the average speeds of successive events confirms that in the no cleaning treatment the ants are indeed modifying their behaviour in response to social information.

The shape of the scaling function, with relatively later periods of acceleration and deceleration, implies a distinguishable but closely related behavioural regime compared with the behaviour inside the nest [7]. We note the consistency between the ‘foraging for work’ model of task allocation in which ants do not specialize on particular tasks [23] and movement patterns that show self-similarity but also flexibility (variable power-law parameters) across a range of physical and social environments. Such an orientation may also have implications for understanding the activity waves that emerge at the level of the colony, which may also be adaptive [15,16]. Further research should be carried out that tracks individual ants (i) performing different tasks inside and outside the nest; and (ii) ants that physically interact outside the nest (rather than simply through chemical deposits) to see how this affects the pattern of results presented here.

Taken together, the findings presented here and in earlier work [7] suggest adjustment of movement durations in response to social information generally occurs between events, and not during them. New social information may be collected during a movement, and processed when at rest, or we suggest, it is both collected and processed when at rest. The corollary of this is that top-down causality from the colony-level of organization does not act continuously on individuals. Intermittent top-down causation from emergent higher organizational levels to their constituent parts (society to the individual; organs to cells) could be an important generic feature for stability in complex systems.

In the eusocial animal system studied here, intermittent response to social information resulting from the motor planning function may be adaptive for both the individual, reducing the burden of information to be processed, and also for the colony, moderating positive feedback effects which may otherwise rapidly propagate social information of poor quality through the system, compromising the effectiveness of the ants’ decentralized task allocation system. If adjustment of movement duration occurs at rest then this may favour more careful collective deliberation. Parallels between social insect systems and other complex systems such as brains have been meaningfully drawn [50,51] and intermittent monitoring of the social environment by individual ants might usefully be compared with analogous phenomena at other organizational levels such as neuron refractory periods (temporary unresponsiveness to further stimulation by other connected neurons after an action potentional is triggered) in future research.

Our findings point to a simple, scalable movement algorithm operating endogenously in a similar manner across all *T. albipennis* workers, one that can be employed in foraging for work both inside and outside the nest. Self-similarity of movement behaviour across differing environments and task allocation problems, and intermittent response to social information, are potentially widespread characteristics of complex adaptive social systems, including human ones; further occurrences may be identified by tracking the movements of organisms across an array of controlled and natural environmental conditions.

## Supplementary Material

Statistical analysis

## Data accessibility

All data can be found on the Dryad data repository (http://dx.doi.org/10.5061/dryad.jk53j).
