# Supplementary material for: Ants determine their next move at rest: motor planning and causality in complex systems
Source: R Soc Open Sci. 2016 Jan 13;3(1):150534. doi: 10.1098/rsos.150534 (PMC4736936; doi:10.1098/rsos.150534)
Supplement: Statistical analysis [file rsos150534supp1.docx]

**ELECTRONIC SUPPLEMENTARY MATERIAL**

**Ants determine their next move at rest, not on the fly**

*Edmund R. Hunt, Roland J. Baddeley, Alan Worley, Ana B. Sendova-Franks, Nigel R. Franks*

***Statistical analysis***

| Section | Description |
| --- | --- |
| Figure S1 | The event velocity threshold $v_{e}$ for the different coarse-graining levels  $\lambda=2, 4, 8, 16$ was determined by reference to the relevant empirical distribution of event speeds. The threshold speed which defines an event is located at the minimum between the peaks of the bimodal distribution, which shifts with $\lambda$. |
| Figure S2 | Testing by colony for the linear relationship between log_10_ (average event speed) and log_10_ (event duration) |
| Table S1 | Parameter values by colony for the linear relationship between log_10_ (average event speed) and log_10_ (event duration).  Also, General Linear Mixed Models (across all colonies) to assess whether the parameters $a$ and $\beta$ in the power-law relationship $\left\langle v(T) \right\rangle_{t}=aT^{\beta}$ are significantly different between the no cleaning and cleaning treatment, at different coarse-graining levels. |
| Table S2 | Results of GLMM analysis for average event durations for ants inside and outside the nest. |
| Table S3 | Results GLMM analysis for the average stopping duration between events for ants inside and outside the nest. |
| Table S4 | Results of GLMM analysis for average event speed for ants inside and outside the nest. |
| Table S5 | The correlation between the average speeds of successive events is tested for different coarse-graining levels. Note that at lower coarse-graining levels, there are more events recorded, hence significance is found with smaller differences between weighted correlations. |
| Table S6 | Comparison of overall event speed correlation between experiments and treatments |
| Table S7 | Table S1 repeated but for log-binning factor $a=1.1$ rather than 1.2 |

Note on coarse-graining levels: considering that most stopping events are of one time step in duration for $\lambda=8$ (figure 4, main paper), or around $0.8s$, we suggest that $\lambda=16$ is an excessive degree of coarse-graining, as it results in time steps of around $1.6s$, in comparison to the original data recorded with ${\Delta t}^{(1)}=0.1\pm0.004s$. As a result, the boundaries of many activity event (periods of stopping) may be averaged out and lost in the analysis for $\lambda=16$, and hence we consider that the results for $\lambda=2, 4, 8$ are more reliable.


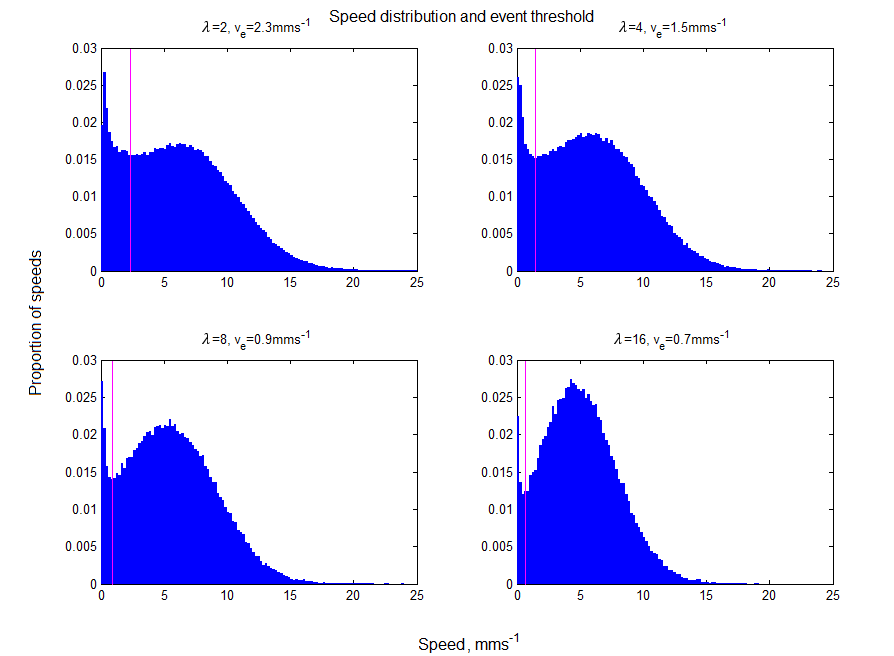


**Figure S1:** The distribution of instantaneous speeds for all 33 ants (15 in the no cleaning treatment, 18 in the cleaning treatment). A bimodality is observed with the first peak near to zero. The event speed threshold $v_{e}$ is determined by reference to the turning point in the distribution, which we take to be the cut-off in discernibility between non-zero and zero speeds.

**
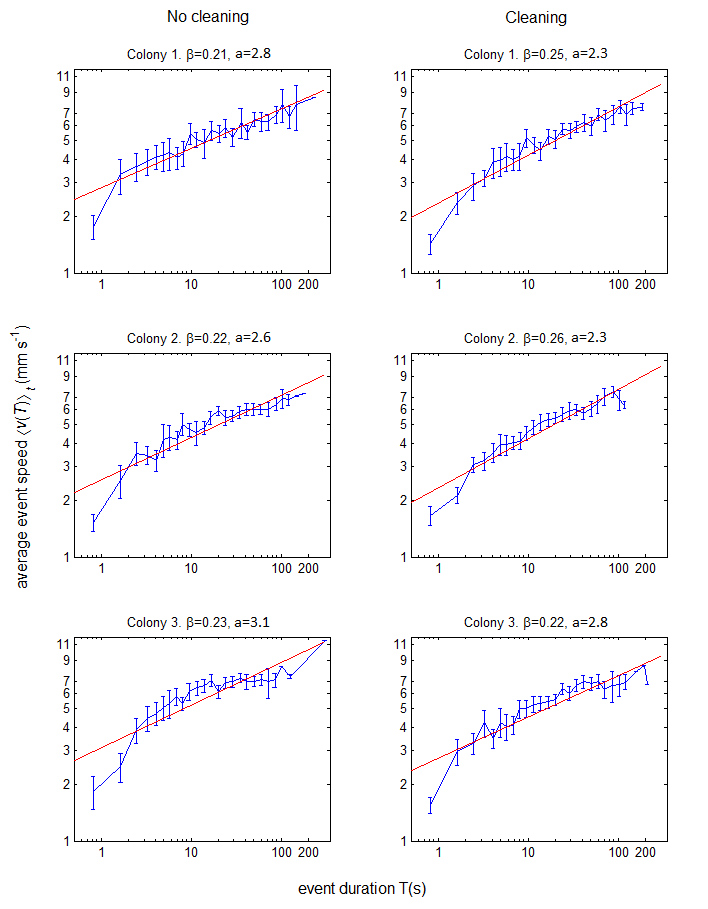
Figure S2**: Linear regression of log_10_ (average event speed $\left\langle v\left( T \right) \right\rangle_{t}$) on log_10_ (event duration $T$) for all 3 colonies and both treatments. Error bars indicate 95% confidence interval. Data points without an error bar originate from one event only. The red line on the log-log plot indicates a power-law relationship $\left\langle v(T) \right\rangle_{t}=aT^{\beta}$, with slope $\beta$ and intercept $a$ at $\log_{10} T=1$.

**Table S1:** by-colony results for the average event speed and duration linear fit, and GLMM result

| $\boldsymbol{\lambda=2}$  0.2s bins up to 1.5s, starting with [0.1 0.3), log bins beyond it starting with [1.5 1.73) (j=2,3)  General linear mixed model result, over all colonies: $\beta$ is not significantly different between NC and C  ($\beta_{NC}=0.201$, $\beta_{C}=0.211$, $t= 0.990$, $n=152$, $p=0.324$).  $a$ is significantly lower in C  ($a_{NC}=5.36$, $a_{C}=5.09$, $t=-2.471$, $n=152$, $p=0.015$)  There is no significant effect from the random factor colony (z = 0.855, p = 0.392).   \| colony \| C \| C \| C \| C \| C \| C \| \| --- \| --- \| --- \| --- \| --- \| --- \| --- \| \| exponent β \| 0.22 \| 0.20 \| 0.18 \| 0.22 \| 0.20 \| 0.22 \| \| 95% CI for β \| ± 0.01 \| ± 0.02 \| ± 0.03 \| ± 0.03 \| ± 0.04 \| ± 0.02 \| \| coefficient *a* \| 5.2 \| 5.1 \| 5.2 \| 4.9 \| 5.6 \| 5.1 \| \| 95% CI for *a* \| ± 0.07 \| ± 0.1 \| ± 0.2 \| ± 0.2 \| ± 0.3 \| ± 0.1 \|   $\boldsymbol{\lambda=4}$  0.4s bins up to 3s, starting with [0.2 0.6), log bins beyond it starting with [3 3.58) (j=6,7)  General linear mixed model result, over all colonies: $\beta$ is not significantly different between NC and C  ($\beta_{NC}=0.217$, $\beta_{C}=0.235$, $t= 1.287$, $n=141$, $p=0.324$).  $a$ is significantly lower in C  ($a_{NC}=3.89$, $a_{C}=3.61$, $t=-1.993$, $n=141$, $p=0.048$)  There is no significant effect from the random factor colony (z = 0.901, p = 0.368).   \| colony \| C \| C \| C \| C \| C \| C \| \| --- \| --- \| --- \| --- \| --- \| --- \| --- \| \| exponent β \| 0.23 \| 0.22 \| 0.19 \| 0.25 \| 0.24 \| 0.24 \| \| 95% CI for β \| ± 0.03 \| ± 0.03 \| ± 0.04 \| ± 0.03 \| ± 0.04 \| ± 0.04 \| \| coefficient *a* \| 3.7 \| 3.7 \| 3.8 \| 3.5 \| 4.1 \| 3.6 \| \| 95% CI for *a* \| ± 0.2 \| ± 0.3 \| ± 0.3 \| ± 0.2 \| ± 0.3 \| ± 0.3 \| |
| --- | --- | --- | --- | --- | --- | --- | --- | --- | --- | --- | --- | --- | --- | --- | --- | --- | --- | --- | --- | --- | --- | --- | --- | --- | --- | --- | --- | --- | --- | --- | --- | --- | --- | --- | --- | --- | --- | --- | --- | --- | --- | --- | --- | --- | --- | --- | --- | --- | --- | --- | --- | --- | --- | --- | --- | --- | --- | --- | --- | --- | --- | --- | --- | --- | --- | --- | --- | --- | --- | --- |

| $\boldsymbol{\lambda=8}$  0.8s bins up to 5.2s, starting with [0.4 1.2), log bins beyond it starting with [5.2 6.19) (j=9,10)  General linear mixed model result, over all colonies: $\beta$ is not significantly different between NC and C  ($\beta_{NC}=0.218$, $\beta_{C}=0.240$, $t= 1.388$, $n=154$, $p=0.167$)  $a$ is significantly lower in C  ($a_{NC}=2.82$, $a_{C}=2.45$, $t=-2.609$, $n=154$, $p=0.010$)  There is no significant effect from the random factor colony (z = 0.909, p = 0.363).   \| colony \| C \| C \| C \| C \| C \| C \| \| --- \| --- \| --- \| --- \| --- \| --- \| --- \| \| exponent β \| 0.21 \| 0.25 \| 0.22 \| 0.26 \| 0.23 \| 0.22 \| \| 95% CI for β \| ± 0.03 \| ± 0.04 \| ± 0.04 \| ± 0.04 \| ± 0.05 \| ± 0.04 \| \| coefficient *a* \| 2.8 \| 2.3 \| 2.6 \| 2.3 \| 3.1 \| 2.8 \| \| 95% CI for *a* \| ± 0.3 \| ± 0.3 \| ± 0.3 \| ± 0.3 \| ± 0.4 \| ± 0.4 \|   $\boldsymbol{\lambda=16}$  1.6s bins up to 8.8s, starting with [0.8 2.4), log bins beyond it starting with [8.8 10.70) (j=12,13)  General linear mixed model result, over all colonies: $\beta$ is not significantly different between NC and C  ($\beta_{NC}=0.227$, $\beta_{C}=0.237$, $t= 0.477$, $n=153$, $p=0.634$)  $a$ is **not** significantly different between NC and C  ($a_{NC}=2.00$, $a_{C}=1.79$, $t=-1.371$, $n=153$, $p=0.172$)  There is no significant effect from the random factor colony (z = 0.874, p = 0.382).   \| colony \| C \| C \| C \| C \| C \| C \| \| --- \| --- \| --- \| --- \| --- \| --- \| --- \| \| exponent β \| 0.24 \| 0.27 \| 0.27 \| 0.24 \| 0.19 \| 0.20 \| \| 95% CI for β \| ± 0.08 \| ± 0.05 \| ± 0.06 \| ± 0.03 \| ± 0.06 \| ± 0.04 \| \| coefficient *a* \| 1.9 \| 1.5 \| 1.6 \| 1.7 \| 2.6 \| 2.1 \| \| 95% CI for *a* \| ± 0.9 \| ± 0.7 \| ± 0.7 \| ± 0.4 \| ± 0.6 \| ± 0.5 \| |
| --- | --- | --- | --- | --- | --- | --- | --- | --- | --- | --- | --- | --- | --- | --- | --- | --- | --- | --- | --- | --- | --- | --- | --- | --- | --- | --- | --- | --- | --- | --- | --- | --- | --- | --- | --- | --- | --- | --- | --- | --- | --- | --- | --- | --- | --- | --- | --- | --- | --- | --- | --- | --- | --- | --- | --- | --- | --- | --- | --- | --- | --- | --- | --- | --- | --- | --- | --- | --- | --- | --- |

**Table S2: Event durations**

Coarse-graining $\lambda=8.$

|  |  |  | 95% confidence interval | |
| --- | --- | --- | --- | --- |
| Experiment | Treatment | Geometric mean event duration, s | Lower | Upper |
| Inside nest | Small | 3.459 | 2.965 | 4.046 |
|  | Large | 3.532 | 2.958 | 3.864 |

Applying a log_10_ transform, geometric mean event duration is not found to be significantly different between nest sizes (n=16,480, t=1.733, p = 0.083); there is no significant effect from the random factor colony on speed (parameter in model is redundant).

|  |  |  | 95% confidence interval | |
| --- | --- | --- | --- | --- |
| Experiment | Treatment | Geometric mean event duration, s | Lower | Upper |
| Outside nest | No clean | 10.520 | 9.354 | 11.830 |
|  | Clean | 8.433 | 6.934 | 10.257 |

Applying a log_10_ transform, the geometric mean event duration is significantly shorter in the cleaning treatment, (n=4289, t=-5.580, p<0.001); there is no significant effect from the random factor colony on speed (z = 0.877, p = 0.381).

Figure 5a from the main text is replicated below, 95% confidence intervals are shown.

**Table S3: Stopping durations**

Coarse-graining $\lambda=8.$

|  |  |  | 95% confidence interval | |
| --- | --- | --- | --- | --- |
| Experiment | Treatment | Geometric mean stopping duration, s | Lower | Upper |
| Inside nest | Smaller | 3.350 | 3.112 | 3.606 |
|  | Larger | 3.750 | 3.334 | 4.207 |

Applying a log_10_ transform, geometric mean stopping duration between events is found to be significantly longer in the larger nest (n=16,421, t=5.105, p < 0.001); there is no significant effect from the random factor colony on speed (z = 0.912, p = 0.362).

|  |  |  | 95% confidence interval | |
| --- | --- | --- | --- | --- |
| Experiment | Treatment | Geometric mean stopping duration, s | Lower | Upper |
| Outside nest | No clean | 1.330 | 1.274 | 1.390 |
|  | Clean | 1.291 | 1.183 | 1.406 |

Applying a log_10_ transform, there is no significant difference in the geometric mean stopping duration between treatments, (n=4256, t=-1.458, p=0.145); there is no significant effect from the random factor colony on speed (z = 0.660, p = 0.510).

Figure 5b from the main text is replicated below, 95% confidence intervals are shown.

**Table S4: Average event speed**

|  |  |  |  | 95% confidence interval | |
| --- | --- | --- | --- | --- | --- |
| Experiment | Treatment | Mean speed mms^-1^ | Std. error | Lower | Upper |
| Inside nest | Smaller | 0.230 | 0.008 | 0.214 | 0.246 |
|  | Larger | 0.288 | 0.013 | 0.262 | 0.313 |

Coarse-graining $\lambda=8.$

|  |  |  |  | 95% confidence interval | |
| --- | --- | --- | --- | --- | --- |
| Experiment | Treatment | Mean speed mms^-1^ | Std. error | Lower | Upper |
| Outside nest | No clean | 5.072 | 0.258 | 4.566 | 5.578 |
|  | Clean | 4.486 | 0.323 | 3.853 | 5.119 |

The mean event speed is significantly higher in the larger nest (n=16,480, t=12.275, p < 0.001); there is no significant effect from the random factor colony on speed (z = 0.912, p = 0.362)

The cleaning treatment has a significantly lower average speed than the no cleaning treatment, (n=4289, t=-9.052, p < 0.001); there is no significant effect from the random factor colony on speed (z = 0.985, p = 0.324).

Figure 5c from the main text is replicated below, 95% confidence intervals are shown.

**Table S5: Correlation analysis of successive average event speeds**

Critical values for non-cleaning (NC) treatment: 5-1=4 d.f., 5% significance: χ^2^_4,0.05(2)_ = 9.488

For cleaning (C) treatment: 6-1=5 d.f., 5% significance: χ^2^_5,0.05(2)_ = 11.070

| $\boldsymbol{\lambda}\mathbf{=2,}\mathbf{v}_{\mathbf{e}}\mathbf{=2.3mm}\mathbf{s}^{\mathbf{-1}}$   \| colony \| C \| C \| C \| C \| C \| C \| \| --- \| --- \| --- \| --- \| --- \| --- \| --- \| \| z chi-square \| 7.8 \| 18.1 \| 27.2 \| 25.2 \| 13.3 \| 16.6 \| \| Weighted z_w_ \| 0.31 \| 0.36 \| 0.29 \| 0.36 \| 0.21 \| 0.28 \| \| Z-score \|  \| -2.09 \|  \| -3.47 \|  \| -3.29 \| \| *p* (2-tailed) \|  \| 0.036 \|  \| 0.0005 \|  \| 0.001 \|   $\boldsymbol{\lambda}\mathbf{=4,}\mathbf{v}_{\mathbf{e}}\mathbf{=1.5mm}\mathbf{s}^{\mathbf{-1}}$   \| colony \| C \| C \| C \| C \| C \| C \| \| --- \| --- \| --- \| --- \| --- \| --- \| --- \| \| z chi-square \| 4.3 \| 14.5 \| 13.7 \| 26.0 \| 5.6 \| 11.6 \| \| Weighted z_w_ \| 0.33 \| 0.50 \| 0.31 \| 0.39 \| 0.20 \| 0.34 \| \| Z-score \|  \| -5.09 \|  \| -2.58 \|  \| -4.32 \| \| *p* (2-tailed) \|  \| 0.0000004 \|  \| 0.01 \|  \| 0.00002 \|   $\boldsymbol{\lambda}\mathbf{=8,}\mathbf{v}_{\mathbf{e}}\mathbf{=0.9mm}\mathbf{s}^{\mathbf{-1}}$   \| colony \| C \| C \| C \| C \| C \| C \| \| --- \| --- \| --- \| --- \| --- \| --- \| --- \| \| z chi-square \| 8.3 \| 28.1 \| 2.7 \| 8.1 \| 4.2 \| 11.2 \| \| Weighted z_w_ \| 0.34 \| 0.54 \| 0.27 \| 0.45 \| 0.15 \| 0.30 \| \| Z-score \|  \| -3.43 \|  \| -3.50 \|  \| -2.89 \| \| *p* (2-tailed) \|  \| 0.0006 \|  \| 0.0005 \|  \| 0.004 \|   $\boldsymbol{\lambda}\mathbf{=16,}\mathbf{v}_{\mathbf{e}}\mathbf{=0.7mm}\mathbf{s}^{\mathbf{-1}}$   \| colony \| C \| C \| C \| C \| C \| C \| \| --- \| --- \| --- \| --- \| --- \| --- \| --- \| \| z chi-square \| 5.0 \| 14.9 \| 8.1 \| 13.7 \| 8.3 \| 8.5 \| \| Weighted z_w_ \| 0.30 \| 0.54 \| 0.26 \| 0.36 \| 0.19 \| 0.20 \| \| Z-score \|  \| -2.25 \|  \| -1.12 \|  \| -0.16 \| \| *p* (2-tailed) \|  \| 0.02 \|  \| 0.26 \|  \| 0.87 \| |
| --- | --- | --- | --- | --- | --- | --- | --- | --- | --- | --- | --- | --- | --- | --- | --- | --- | --- | --- | --- | --- | --- | --- | --- | --- | --- | --- | --- | --- | --- | --- | --- | --- | --- | --- | --- | --- | --- | --- | --- | --- | --- | --- | --- | --- | --- | --- | --- | --- | --- | --- | --- | --- | --- | --- | --- | --- | --- | --- | --- | --- | --- | --- | --- | --- | --- | --- | --- | --- | --- | --- | --- | --- | --- | --- | --- | --- | --- | --- | --- | --- | --- | --- | --- | --- | --- | --- | --- | --- | --- | --- | --- | --- | --- | --- | --- | --- | --- | --- | --- | --- | --- | --- | --- | --- | --- | --- | --- | --- | --- | --- | --- | --- | --- | --- | --- | --- | --- | --- | --- | --- | --- | --- | --- | --- | --- | --- | --- | --- | --- | --- | --- | --- | --- | --- | --- | --- | --- | --- | --- | --- |

**Table S6: Comparison of overall event speed correlation between experiments and treatments**

| \|  \| Inside \| Inside \| Outside \| Outside \| \| --- \| --- \| --- \| --- \| --- \| \|  \| Smaller \| Larger \| No clean \| Clean \| \| $z_{w}$ \| 0.283 \| 0.338 \| 0.238 \| 0.432 \| \| $\sum(n_{i}-3)$ \| 8419 \| 7366 \| 1653 \| 2486 \| \| Standard error in $z_{w}$ \| 0.011 \| 0.012 \| 0.025 \| 0.020 \| \| $r_{w}$ \| 0.278 \| 0.326 \| 0.234 \| 0.407 \| \|  \|  \|  \|  \|  \|   The weighted $z_{w}$ value for the outside, cleaning treatment is significantly higher than the other 3 treatments. The outside, no cleaning treatment $z_{w}$ is not statistically different from the inside, smaller nest treatment, but it is lower than the inside, larger nest treatment. The inside, larger nest treatment has a significantly higher $z_{w}$ than the inside, smaller nest treatment.   \| **Z values** \| In, smaller \| In, larger \| Out, NC \| Out, C \| \| --- \| --- \| --- \| --- \| --- \| \| In, smaller \|  \| 3.446 \| -1.666 \| 6.551 \| \| In, larger \|  \|  \| -3.665 \| 4.077 \| \| Out, NC \|  \|  \|  \| 6.122 \| \| Out, C \|  \|  \|  \|  \|   The critical value is $Z_{0.05\left( 2 \right)}$ = 1.960 |
| --- | --- | --- | --- | --- | --- | --- | --- | --- | --- | --- | --- | --- | --- | --- | --- | --- | --- | --- | --- | --- | --- | --- | --- | --- | --- | --- | --- | --- | --- | --- | --- | --- | --- | --- | --- | --- | --- | --- | --- | --- | --- | --- | --- | --- | --- | --- | --- | --- | --- | --- | --- | --- | --- | --- | --- | --- | --- | --- | --- | --- |

Figure 9 from the main text is replicated below, 95% confidence intervals are shown.

**Table S7:** average event speed and duration linear fit, and GLMM result, for $\boldsymbol{a=1.1}$ in binning method $[a^{j} a^{j+1})$ Compare with table S1, numbers similar and statistical findings similar, except significance **is** found in the difference between intercepts for $\lambda=16$, which is in the same direction as before (lower in cleaning treatment).

| $\boldsymbol{n=2}$  0.2s bins up to 2.1s, starting with [0.1 0.3), log bins beyond it starting with [2.1 2.36) (j=8,9)  General linear mixed model result, over all colonies: $\beta$ is not significantly different between NC and C  ($\beta_{NC}=0.192$, $\beta_{C}=0.197$, $t= 0.554$, $n=239$, $p=0.580$).  $a$ is significantly lower in C  ($a_{NC}=5.47$, $a_{C}=5.26$, $t=-2.047$, $n=239$, $p=0.042$)  There is no significant effect from the random factor colony (z = 0.929, p = 0.353).   \| colony \| C \| C \| C \| C \| C \| C \| \| --- \| --- \| --- \| --- \| --- \| --- \| --- \| \| exponent β \| 0.21 \| 0.19 \| 0.17 \| 0.22 \| 0.20 \| 0.21 \| \| 95% CI for β \| ± 0.01 \| ± 0.02 \| ± 0.03 \| ± 0.02 \| ± 0.03 \| ± 0.02 \| \| coefficient *a* \| 5.3 \| 5.3 \| 5.4 \| 5.1 \| 5.8 \| 5.3 \| \| 95% CI for *a* \| ± 0.1 \| ± 0.1 \| ± 0.2 \| ± 0.1 \| ± 0.2 \| ± 0.1 \|   $\boldsymbol{n=4}$  0.4s bins up to 4.6s, starting with [0.2 0.6), log bins beyond it starting with [4.6 5.05) (j=16,17)  General linear mixed model result, over all colonies: $\beta$ is not significantly different between NC and C  ($\beta_{NC}=0.205$, $\beta_{C}=0.221$, $t= 1.423$, $n=242$, $p=0.156$).  $a$ is significantly lower in C  ($a_{NC}=4.07$, $a_{C}=3.80$, $t=-2.283$, $n=242$, $p=0.023$)  There is no significant effect from the random factor colony (z = 0.901, p = 0.342).   \| colony \| C \| C \| C \| C \| C \| C \| \| --- \| --- \| --- \| --- \| --- \| --- \| --- \| \| exponent β \| 0.22 \| 0.21 \| 0.18 \| 0.24 \| 0.22 \| 0.22 \| \| 95% CI for β \| ± 0.02 \| ± 0.03 \| ± 0.03 \| ± 0.03 \| ± 0.03 \| ± 0.03 \| \| coefficient *a* \| 3.8 \| 3.9 \| 4.0 \| 3.6 \| 4.4 \| 3.9 \| \| 95% CI for *a* \| ± 0.2 \| ± 0.2 \| ± 0.3 \| ± 0.2 \| ± 0.2 \| ± 0.2 \| |
| --- | --- | --- | --- | --- | --- | --- | --- | --- | --- | --- | --- | --- | --- | --- | --- | --- | --- | --- | --- | --- | --- | --- | --- | --- | --- | --- | --- | --- | --- | --- | --- | --- | --- | --- | --- | --- | --- | --- | --- | --- | --- | --- | --- | --- | --- | --- | --- | --- | --- | --- | --- | --- | --- | --- | --- | --- | --- | --- | --- | --- | --- | --- | --- | --- | --- | --- | --- | --- | --- | --- |

| $\boldsymbol{n=8}$  0.8s bins up to 10s, starting with [0.4 1.2), log bins beyond it starting with [10 10.83) (j=24,25)  General linear mixed model result, over all colonies: $\beta$ is not significantly different between NC and C  ($\beta_{NC}=0.204$, $\beta_{C}=0.226$, $t= 1.690$, $n=249$, $p=0.092$)  $a$ is significantly lower in C  ($a_{NC}=2.97$, $a_{C}=2.61$, $t=-2.960$, $n=249$, $p=0.003$)  There is no significant effect from the random factor colony (z = 0.950, p = 0.342).   \| colony \| C \| C \| C \| C \| C \| C \| \| --- \| --- \| --- \| --- \| --- \| --- \| --- \| \| exponent β \| 0.20 \| 0.24 \| 0.21 \| 0.24 \| 0.20 \| 0.21 \| \| 95% CI for β \| ± 0.03 \| ± 0.03 \| ± 0.03 \| ± 0.03 \| ± 0.04 \| ± 0.03 \| \| coefficient *a* \| 2.9 \| 2.5 \| 0.41 \| 2.4 \| 3.4 \| 2.9 \| \| 95% CI for *a* \| ± 0.3 \| ± 0.3 \| ± 0.05 \| ± 0.3 \| ± 0.4 \| ± 0.3 \|   $\boldsymbol{n=16}$  1.6s bins up to 23.2s, starting with [0.8 2.4), log bins beyond it starting with [23.2 25.55) (j=33,34)  General linear mixed model result, over all colonies: $\beta$ is not significantly different between NC and C  ($\beta_{NC}=0.206$, $\beta_{C}=0.2$36, $t= 1.537$, $n=243$, $p=0.126$)  $a$ **is** significantly lower in C  ($a_{NC}=2.19$, $a_{C}=1.81$, $t=-2.489$, $n=243$, $p=0.014$)  There is no significant effect from the random factor colony (z = 0.943, p = 0.346).   \| colony \| C \| C \| C \| C \| C \| C \| \| --- \| --- \| --- \| --- \| --- \| --- \| --- \| \| exponent β \| 0.21 \| 0.27 \| 0.24 \| 0.22 \| 0.17 \| 0.21 \| \| 95% CI for β \| ± 0.07 \| ± 0.04 \| ± 0.04 \| ± 0.03 \| ± 0.05 \| ± 0.04 \| \| coefficient *a* \| 2.1 \| 1.5 \| 1.8 \| 1.8 \| 2.8 \| 2.1 \| \| 95% CI for *a* \| ± 0.8 \| ± 0.6 \| ± 0.5 \| ± 0.4 \| ± 0.5 \| ± 0.4 \| |
| --- | --- | --- | --- | --- | --- | --- | --- | --- | --- | --- | --- | --- | --- | --- | --- | --- | --- | --- | --- | --- | --- | --- | --- | --- | --- | --- | --- | --- | --- | --- | --- | --- | --- | --- | --- | --- | --- | --- | --- | --- | --- | --- | --- | --- | --- | --- | --- | --- | --- | --- | --- | --- | --- | --- | --- | --- | --- | --- | --- | --- | --- | --- | --- | --- | --- | --- | --- | --- | --- | --- |
